# Heat Shock Protein 60, Insights to Its Importance in *Histoplasma capsulatum*: From Biofilm Formation to Host-Interaction

**DOI:** 10.3389/fcimb.2020.591950

**Published:** 2021-01-22

**Authors:** Nathália Ferreira Fregonezi, Lariane Teodoro Oliveira, Junya de Lacorte Singulani, Caroline Maria Marcos, Claudia Tavares dos Santos, Maria Lucia Taylor, Maria José Soares Mendes-Giannini, Haroldo Cesar de Oliveira, Ana Marisa Fusco-Almeida

**Affiliations:** ^1^ Department of Clinical Analysis, School of Pharmaceutical Sciences, São Paulo State University-UNESP, Araraquara, Brazil; ^2^ Unidad de Micología, Departamento de Microbiología y Parasitología, Facultad de Medicina, UNAM—Universidad Nacional Autónoma de México, Mexico City, Mexico

**Keywords:** histoplasmosis, biofilm, Hsp60, adhesins, *Galleria mellonella*

## Abstract

Heat shock proteins (Hsps) are among the most widely distributed and evolutionary conserved proteins, acting as essential regulators of diverse constitutive metabolic processes. The Hsp60 of the dimorphic fungal *Histoplasma capsulatum* is the major surface adhesin to mammalian macrophages and studies of antibody-mediated protection against *H. capsulatum* have provided insight into the complexity involving Hsp60. However, nothing is known about the role of Hsp60 regarding biofilms, a mechanism of virulence exhibited by *H. capsulatum*. Considering this, the present study aimed to investigate the influence of the Hsp60 on biofilm features of *H. capsulatum*. Also, the non-conventional model *Galleria mellonella* was used to verify the effect of this protein during *in vivo* interaction. The use of invertebrate models such as *G. mellonella* is highly proposed for the evaluation of pathogenesis, immune response, virulence mechanisms, and antimicrobial compounds. For that purpose, we used a monoclonal antibody (7B6) against Hsp60 and characterized the biofilm of two *H. capsulatum* strains by metabolic activity, biomass content, and images from scanning electron microscopy (SEM) and confocal laser scanning microscopy (CLSM). We also evaluated the survival rate of *G. mellonella* infected with both strains under blockage of Hsp60. The results showed that mAb 7B6 was effective to reduce the metabolic activity and biomass of both *H. capsulatum* strains. Furthermore, the biofilms of cells treated with the antibody were thinner as well as presented a lower amount of cells and extracellular polymeric matrix compared to its non-treated controls. The blockage of Hsp60 before fungal infection of *G. mellonella* larvae also resulted in a significant increase of the larvae survival compared to controls. Our results highlight for the first time the importance of the Hsp60 protein to the establishment of the *H. capsulatum* biofilms and the *G. mellonella* larvae infection. Interestingly, the results with Hsp60 mAb 7B6 in this invertebrate model suggest a pattern of fungus-host interaction different from those previously found in a murine model, which can be due to the different features between insect and mammalian immune cells such as the absence of Fc receptors in hemocytes. However further studies are needed to support this hypothesis

## Introduction

*Histoplasma capsulatum* is a dimorphic pathogenic fungus that causes histoplasmosis, one of the most common pulmonary mycosis in the United States (US) (Armstrong et al., 2018; Maiga et al., 2018; Salzer et al., 2018). Despite endemic in certain areas of the US (e.g. Ohio and Mississippi river valleys), histoplasmosis has a worldwide distribution and is also one of the top AIDS-defining conditions and AIDS-related causes of death in Latin America (Adenis et al., 2018; Papalini et al., 2019).

The histoplasmosis infection occurs *via* inhalation of conidial spores that transform into yeasts within the mammalian host (Mittal et al., 2019). As a facultative intracellular fungus, *H. capsulatum* yeasts are readily phagocytosed by resident macrophages, where they survive and replicate. During the early phases of infection, alveolar macrophages (Mφ) recognize unopsonized *H. capsulatum* yeasts and microconidia *via* the CD18 family of adhesion-promoting glycoproteins, LFA-1 (CD11a/CD18), complement receptor 3 (CR3; CD11b/CD18), and CR4 (CD11c/CD18) (Bullock and Wright, 1987; Newman et al., 1990).

The adhesion capacity to host tissue is important to several microorganisms, and a relevant mechanism of virulence is described to dimorphic fungi (McMahon et al., 1995; Brandhorst and Klein, 2000; Marcos et al., 2016; Portuondo et al., 2016). However, the interaction between host-pathogen is not the only factor involved in the infectious process, but also the cell-cell interaction/adhesion. The adhesion is also crucial for the formation of resistance structures, called biofilms (Verstrepen and Klis, 2006; Borges et al., 2018). Like many other pathogenic fungi, *H. capsulatum* yeasts can form biofilms *in vitro* (Pitangui et al., 2012; Gonçalves et al., 2020). Biofilms are defined as a dynamic community of microorganisms strongly linked with each other and attached to a biotic or abiotic surface, surrounded by a self-produced extracellular polymeric matrix (EPM) that provides protection against hostile environments and is also related to reduced antifungal activity (Costerton et al., 1995; Baillie and Douglas, 2000; Brilhante et al., 2015; Zarnowski et al., 2018). *In vivo H. capsulatum* biofilms has never been proved. However, the *H. capsulatum* yeasts can adhere to various cryopreserved bat organs, such as lung, spleen, liver, and intestine (Suarez-Alvarez et al., 2010), human epithelial cell lines (Pitangui et al., 2012) and also, to endothelium and prosthetic valves (Ledtke et al., 2012; Lorchirachonkul et al., 2013; Riddell et al., 2014).

The adhesion process could be mediated by several surface-associated proteins. In *H. capsulatum*, one of these proteins is the heat shock protein 60 (Hsp60), responsible for the adhesion and interaction with CD11b/CD18 (CR3) Mφ receptor (Long et al., 2003), therefore playing an essential role in the infection process.

Heat shock proteins (HSPs) are ubiquitously expressed, highly conserved proteins, known to act as molecular chaperones with important functions, such as the transport of proteins and promotion of folding and assembly of polypeptides in fungi (Kubota et al., 1995; Leach et al., 2012; Cleare et al., 2017). In addition to its intracellular biologic activities, Hsp60 is a prominent target of the humoral and cellular immune response to *H. capsulatum* (Gomez A. M. et al., 1991; Gomez F. J. et al., 1991). *H. capsulatum* Hsp60 was first identified as a 62-kDa protein isolated from the cell wall and membrane extract and showed antigenic (Gomez A. M. et al., 1991) and immunogenic (Gomez et al., 1995) properties.

Hsp60 is reported to be predominantly in the cytosolic fraction of cells (Kubota et al., 1995; Kalderon et al., 2015). However, to act as a ligand for the host cell, in *H. capsulatum*, Hsp60 is expressed in clusters on the cell wall (Long et al., 2003), promoting recognition, adhesion, and phagocytosis of the fungi. The Hsp60/CR3 interaction results in phagocytosis without complete activation of the phagocyte, leading to a non-inflammatory immunological response (Wolf et al., 1987; Ehlers, 2000; Lin et al., 2010; Mihu and Nosanchuk, 2012).

It is of great knowledge that *H. capsulatum* can infect mammals, and murine models are traditionally used for the study of this fungal virulence. However, it is also known that *H. capsulatum* is capable of infect *G. mellonella* larvae (Thomaz et al., 2013), making this non-conventional animal model an important tool to understand *Histoplasma*-host interaction. Invertebrate animals have emerged as alternative models to mammals because breeding is simple and inexpensive (Fuchs and Mylonakis, 2006; Binder et al., 2016). In this aspect, the study of the pathogenesis of different microorganism including dimorphic fungi such as *Paracoccidioides* spp., *Sporothrix* spp., *Talaromyces marneffei* (*Penicillium marneffei*), and *H. capsulatum* has been evaluated in *G. mellonella* larvae (Thomaz et al., 2013; Huang et al., 2015; Scorzoni et al., 2015; Clavijo-Giraldo et al., 2016). The model is especially advantageous for dimorphic fungi due to the possibility that the larvae are kept at 37°C during survival assays and they present six types of immune cells called hemocytes, which have structural and functional similarities to cells of the mammalian immune system (Singulani et al., 2018).

Our current work sought to understand the importance of Hsp60 in *H. capsulatum* biofilms formation and the fungi virulence in the alternative model *G. mellonella*, gaining new insights to a better understanding of cell biology and a future possible application of this protein as a target to therapeutic approaches to histoplasmosis management.

## Materials and Methods

### *Histoplasma capsulatum* Strains and Growth Conditions

*H. capsulatum* strains used in this study included G186A (ATCC 26029), representative of chemotype II, and EH-315. EH-315 was isolated from the intestine of infected bats captured in a cave in the state of Guerrero (Mexico) and is designated by Teixeira et al. (2016) as belonging to a bat-associated species-specific clade (BAC1). EH-315 is deposited in the *H. capsulatum* Culture Collection of the Fungal Immunology Laboratory of the Department of Microbiology and Parasitology, from the School of Medicine, National Autonomous University of Mexico (UNAM) (www.histoplas-mex.unam.mx), which is registered in the database of the World Data Centre for Microorganisms (WDCM) with number LIH-UNAM WDCM817. The G186A is classified as H81 human lineage (Kasuga et al., 2003). Both strains are now deposited in the collection of strains at the Clinical Mycology Laboratory of the Faculty of Pharmaceutical Sciences, UNESP (Brazil), and maintained at 37°C in Brain Heart Infusion agar supplemented with 1% of glucose and 0.1% of L-cysteine. Before the experiments, *H. capsulatum* was cultivated in *Histoplasma-*macrophage medium (HMM), composed of HAM-F12 (Sigma) medium, supplemented with glucose (18.2 g/L), glutamic acid (1g/L), HEPES (6 g/L), and L-cysteine (8.4 mg/L) at 37°C and 150 rpm for 48 h.

### *H. capsulatum* Viability After Treatment With Hsp60 mAb (7B6)

Yeast cells were cultured for 48 h in HMM at 37°C and 150 rpm. The cultures were centrifuged at 1000 ×*g* for 10 min, and the pellets were washed three times with phosphate-buffered saline (PBS). To evaluate the viability of *H. capsulatum* yeasts after treatment with Hsp60 mAb 7B6, 10^7^ yeast cells were incubated with 10 μg/ml of Hsp60 mAb 7B6, unspecific IgG (Control IgG) in PBS or PBS alone for 1 h at 37°C. After incubation, the cells were washed with PBS and the cell viability was assessing in a hemocytometer using Trypan blue solution. The Hsp60 mAb 7B6 was gently provided by Dr. Joshua D. Nosanchuk from Albert Einstein College of Medicine. Two independent experiments were performed.

### Immunofluorescence of the Hsp60

After 48 h cultivation in HMM at 37°C and 150 rpm, the *H. capsulatum* culture was centrifuged at 1,000 ×*g* for 10 min, and the pellet washed three times with phosphate-buffered saline (PBS). The yeast cells were fixed with paraformaldehyde 4% and counted with a hemacytometer. Aliquots containing 10^6^ yeast cells were incubated in blocking solution [1% Bovine Serum Albumin (BSA)] for 4 h at 37°C. After washing three times with PBS-Tween 20 0.05% the yeasts were incubated with 10 μg/ml of Hsp60 mAb 7B6 or unspecific IgG (Control IgG) in blocking solution for 1 h at 37°C. Then, after three washes as previously described, yeast cells were incubated for 1 h at 37°C with Alexa Fluor 594-labeled goat anti-mouse IgG (Thermo Fisher Scientific) at a 1:1,000 dilution in blocking solution. After three washes, cells were incubated with fluorescein isothiocyanate (FITC) (Sigma) at 0.5 mg/ml for 45 min at room temperature. Then, the cells were washed and examined with a Zeiss LSM 800 confocal microscope (School of Dentistry of Araraquara, Unesp). Three independent experiments were performed.

### Exploring the Involvement of Hsp60 in *H. capsulatum* Biofilm Formation

#### Biofilm Development

The biofilm formation was performed as described by Gonçalves et al. (2020). To test the influence of *H. capsulatum* Hsp60 in the biofilm development, the protein was blocked through the treatment with the Hsp60 mAb 7B6. To this, after 48 h growth on HMM, the cells were washed three times with PBS and 10^7^ yeast cells were incubated with 10 μg/ml of Hsp60 mAb 7B6 or unspecific IgG (Control IgG) in PBS for 1 h at 37°C. After incubation, the cells were washed with PBS and the fungal suspensions were prepared in sterile PBS at 5×10^6^ cells/ml. Then, 200 μl and 1,000 μl of the inoculum was added to 96-well and 24-well plates, respectively, and incubated at 37°C for 12 h for biofilm pre-adhesion. After pre-adhesion, the supernatant was removed and the wells were washed carefully to remove non-adherent cells. Then, 200 μl and 2,000 μl of HMM medium were added to 96-well and 24-well plates, respectively, and incubated until 144 h. The *H. capsulatum* biofilms were characterized by measuring the biofilm biomass (crystal violet) and metabolic activity (XTT). At the structural level, the biofilms were analyzed by Scanning Electron Microscopy (SEM) and Confocal Laser Scanning Microscopy (CLSM). Non-treated *H. capsulatum* and the yeasts treated with unspecific IgG (Control IgG) were used as controls. The tests described above were repeated three times.

#### Crystal Violet Assay

The biomass quantification was performed by the crystal violet assay in 96-well plates as described by Costa-Orlandi et al. (2014). After 144 h of biofilm formation, the supernatant was removed and the biofilms were washed carefully to remove non-adherent cells. Then, the biofilms were fixed with 200 µl of 100 % methanol for 15 min. After removing the methanol, the wells were left to dry at room temperature. Afterward, 200 µl of 0.1 % crystal violet solution was added and incubated for 20 min. The wells were then washed with distilled water three times and 200 µl of a 33 % solution of acetic acid was added. Subsequently, the content of each plate was transferred to another plate for immediate spectrophotometric reading at 590 nm.

#### XTT Assay

Metabolic activity was evaluated by the XTT (2,3-Bis-(2-Methoxy-4-Nitro-5-Sulfophenyl)-2H-Tetrazolium-5-Carboxanilide) (Sigma) assay in 96-well plates (Martinez and Casadevall, 2007). After 144 h of biofilm formation, the supernatant was removed and the biofilms were washed carefully to remove non-adherent cells. Therefore, 50 μl of XTT solution at 1 mg/ml and 4 µl of menadione solution at 1 mM were added. The plates were incubated at 37°C for 3 to 4 h. The content of each plate was transferred to another plate and spectrophotometric read at 490 nm. To the establishment of the kinetics curve, the XTT-menadione solution was added at 12, 24, 48, 72, 96, 120, 144, and 168 h time points, and four independent experiments were performed.

#### Scanning Electron Microscopy

The topography of the biofilms was assessed by SEM and samples were processed as described by Gonçalves et al. (2020). Biofilms were formed in 24-well plates as described above. After 144 h of biofilm formation, the supernatant was removed and the biofilms were washed with PBS to remove non-adherent cells. Biofilms were then fixed with 2.5% of glutaraldehyde solution (Sigma-Aldrich) for 24 h at 4°C. After fixation, the biofilms were washed with PBS and sequentially dehydrated using ethanol solutions (ranging from 20% to 100%) at room temperature. All samples were dried in a pyrex glass vacuum desiccator. Once dried, the wells were cut using a flame-heated scalpel. Subsequently, the samples were mounted on aluminum and silver cylinders and disposed of in a high vacuum evaporator for gold coating. Topographic images of biofilms were captured under the scanning electron microscope JEOL JSM- 6610LV (School of Dentistry of Araraquara, UNESP).

#### Confocal Laser Scanning Microscopy

Biofilms were formed in 24-well plates as described above. After 144 h of biofilm formation, the supernatant was removed and the biofilm was gently washed with PBS. Live/dead staining was performed using the LIVE/DEAD™ FungaLight™ Yeast Viability Kit (Thermo Fisher Scientific) by incubating the biofilms for 30 min at 37°C with 3.34 μM of the green-fluorescent nucleic acid stain SYTO 9 combined with 20 μM of the red-fluorescent nucleic acid stain propidium iodide (PI) in PBS. Then, the biofilms were washed with PBS, fixed with paraformaldehyde 4% for 24 h, and analyzed with a Zeiss LSM 800 confocal microscope (School of Dentistry of Araraquara, UNESP).

### Survival Assay Using the Alternative Animal Model *Galleria mellonella*

Survival assay was performed according to Thomaz et al. (2013), with modifications. *G. mellonella* larvae (School of Pharmaceutical Sciences, São Paulo State University - UNESP) with a body weight ranging from 150 and 200 mg were randomly chosen for the experiments. Ten larvae per group were kept in Petri dishes at 37°C overnight before use. The inoculum of both *H. capsulatum* strains was prepared in PBS at 1x10^8^ yeasts/ml. To test the influence of Hsp60 in the interaction with the larvae, the inoculum was previously treated with 10 μg/ml of the Hsp60 mAb 7B6 for 1 h at 37°C. Then, the yeasts were washed and suspended in PBS. For each group, larvae were injected with 1x10^6^ yeasts/larvae using a 10 µl Hamilton syringe. Larvae inoculated with non-treated *H. capsulatum* strains, treated with unspecific IgG (Control IgG), and larvae inoculated with sterile PBS were used as controls. All larvae were placed in sterile Petri dishes and maintained in the dark at 37°C. Mortality was monitored for up to 10 days of infection. Larvae were considered dead when they displayed no movement in response to touch. Three independent experiments were performed.

### Statistical Analysis

All data were subjected to statistical analysis using the software GraphPad Prism 5.0 (GraphPad Software, Inc., San Diego, CA). Unless otherwise noted, results were presented as mean ± standard deviation (SD), and compared by analysis of variance (ANOVA) followed by Bonferroni or Tukey tests. Survival curves of *G. mellonella* larvae were plotted as Kaplan–Meier survival curves and compared using log-rank tests. Statistical significance was considered when p < 0.05.

## Results

### *H. capsulatum* Viability After Treatment With Hsp60 mAb 7B6

To evaluate the influence of the Hsp60 mAb 7B6 in *H. capsulatum* viability, the yeast cells were incubated with 10µg/ml of the mAb 7B6 and also with unspecific IgG and PBS for 1 h at 37°C After the treatments, all the conditions showed viabilities higher than 90% (**Table 1**).

**Table 1:** Viability of G186A and EH-315 strains of *Histoplasma capsulatum* after incubation with Hsp60 mAb 7B6.

	G186A	EH-315
	Mean ± SD	Mean ± SD
Hsp60 mAb 7B6	91.34 ± 1.31	91.28 ± 1.92
Control IgG	90.58 ± 1.84	92.99 ± 0.61
PBS	92.06 ± 2.00	92.34 ± 0.40

Results are representative of two independent experiments and values expressed as mean ± SD. Cell viability after 1 h incubation with phosphate-buffered saline (PBS), unspecific IgG (Control IgG) (10 µg/ml) and Hsp60 mAb 7B6 (10 µg/ml) at 37°C and 1,500 rpm.

### Hsp60 Localization by Immunofluorescence

**Figure 1:**
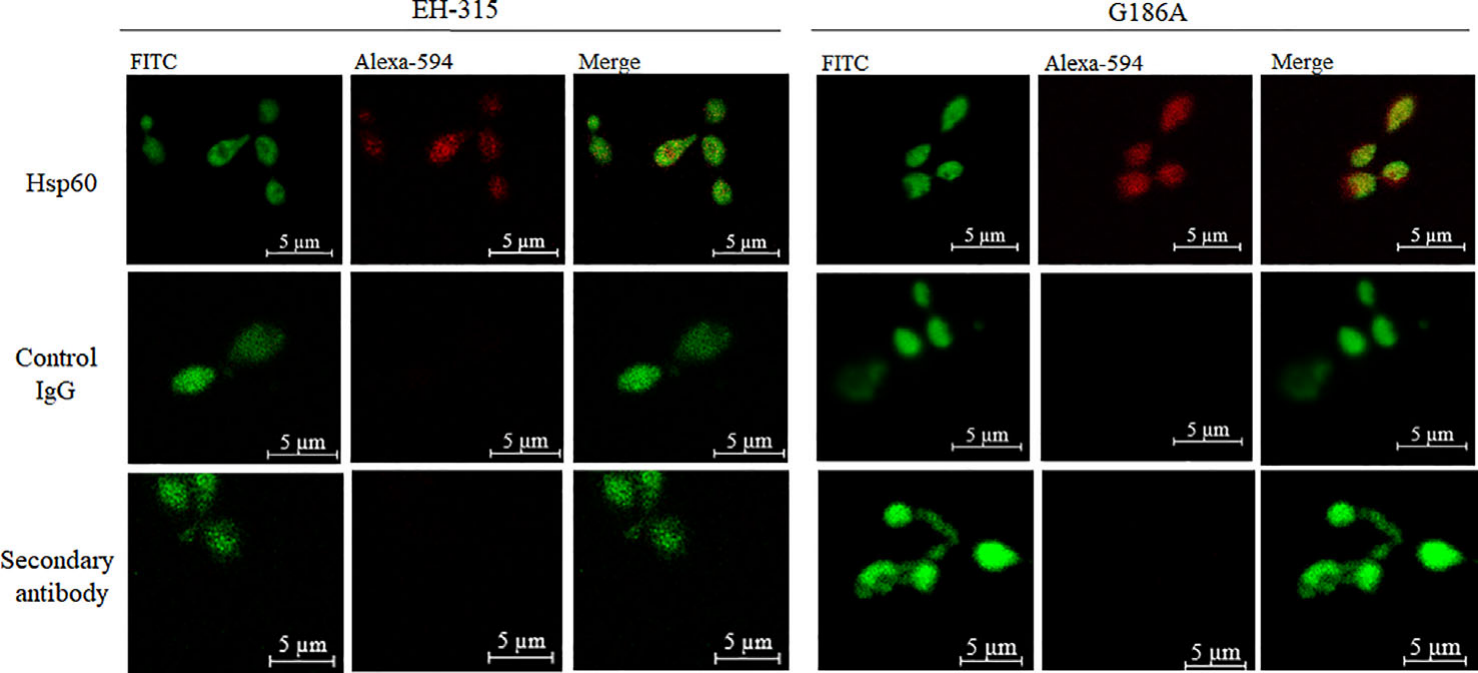
Confocal laser scanning microscopy (CLSM) of mAb-labeled Hsp60 in *H. capsulatum*: immunofluorescence showing labeling of the *H. capsulatum* Hsp60 in EH-315 and G186A strains at 63x. Alexa 594: conjugated with Hsp60 mAb 7B6 or unspecific IgG (Control IgG).

To localize the binding of Hsp60 mAb 7B6 to G186A and EH-315 Hsp60, we assessed the interactions of the mAbs with yeast cells by fluorescence microscopy. mAb 7B6 revealed a diffusion distribution of the Hsp60 in both EH-315 and G186A (**Figure 1**) *H. capsulatum* strains, similar to the previous report of Guimaraes et al. (2009) in G217B strain. Controls with unspecific IgG and secondary antibody were added and showed no signal.

### Kinetic of *H. capsulatum* Biofilm Formation

**Figure 2:**
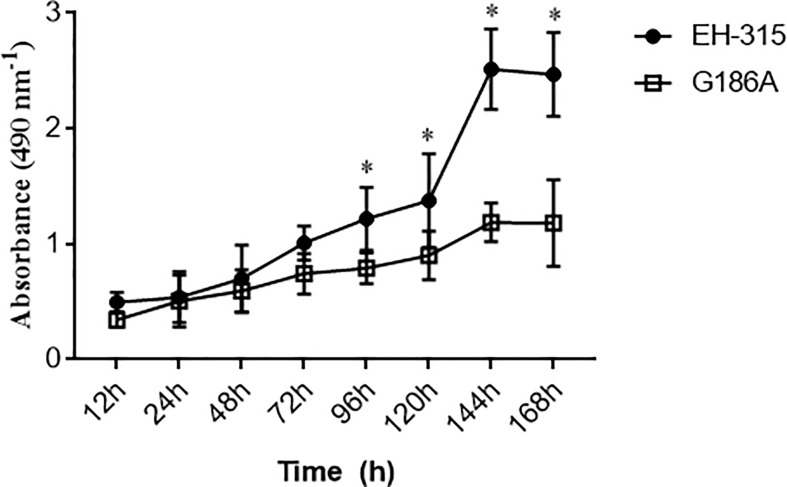
Kinetics of *H. capsulatum* biofilm formation on polystyrene microtiter plates. The metabolic activity of EH-315 and G186A strains was evaluated by the colorimetric XTT reduction assay. Data are representative of four independent experiments and values expressed as mean ± SD. Statistically significant differences between the strains at 96, 120, 144, and 168 h. *p < 0.05.

**Figure 3:**
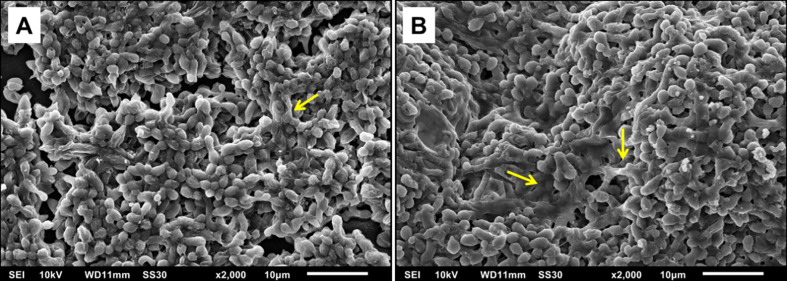
Scanning electron microscopy (SEM) showing yeast adhered to the polystyrene plate indicating the formation of mature biofilms. **(A)** Biofilm of G186A strain at 2000x. **(B)** Biofilm of EH-315 strain at 2000x. The yellow arrows indicate the extracellular polymeric matrix (EPM).

Before evaluating the influence of Hsp60 on *H. capsulatum* biofilm formation, the XTT assay and SEM analysis were performed with both G186A and EH-315 strains to establish a kinetic curve of the biofilm formation (**Figure 2**) and to evaluate the biofilm structure (**Figure 3**), respectively. The metabolic activity of the biofilms increased over time and the highest growth was observed in the period from 72 to 144 h. Both strains produced consistent and mature biofilms at 144 h, reaching the plateau between 144 and 168 h (**Figure 2**).

The kinetics of biofilm formation was similar for both strains during the initial steps, but after 96 h there was a statistically significant difference (P<0.005) between the strains, with EH-315 presenting higher metabolic activity (**Figure 2**). Considering that biofilm maturation occurs in 144 h (**Figure 2**), SEM analysis of G186A (**Figure 3A**) and EH-315 (**Figure 3B**) strains were performed at this time and showed numerous *H. capsulatum* yeasts firmly adhered to the plastic surface and embedded in an EPM.

### Influence of Hsp60 on *H. capsulatum* Biofilms

Quantitative measurement of biofilms formed on polystyrene microtiter plates following incubation for 144 h was performed using crystal violet staining and XTT reduction assay, as previously described. Corroborating with the findings during XTT kinetics, the environmental *H. capsulatum* EH-315 strain presented higher metabolic activity (p<0.05) and also formed a more robust biofilm, with higher biomass content compared to the human *H. capsulatum* G186A strain.

**Figure 4:**
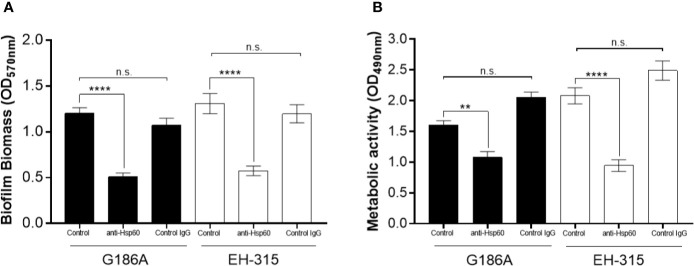
Influence of Hsp60 in *H. capsulatum* EH-315 and G186A biofilm formation. **(A)** Quantification of total biomass by crystal violet staining and **(B)** quantification of metabolic activity by XTT reduction assay. Data are representative of three independent experiments and values expressed as mean ± SEM. **p < 0.01; ****p < 0.0001; n.s., not significant.

The pre-treatment of yeasts with Hsp60 mAb 7B6 resulted in the formation of a thin biofilm, with reduced biomass to both strains (p<0.0001) (**Figure 4A**). Also, both G186A (p<0.05) and EH-315 (p<0.0001) biofilms of pre-treated yeasts presented significantly reduced metabolic activity compared to its non-treated controls (**Figure 4B**). The pre-treatment of both strains with control IgG did not alter the biomass nor the metabolic activity compared to those of untreated fungi.

**Figure 5:**
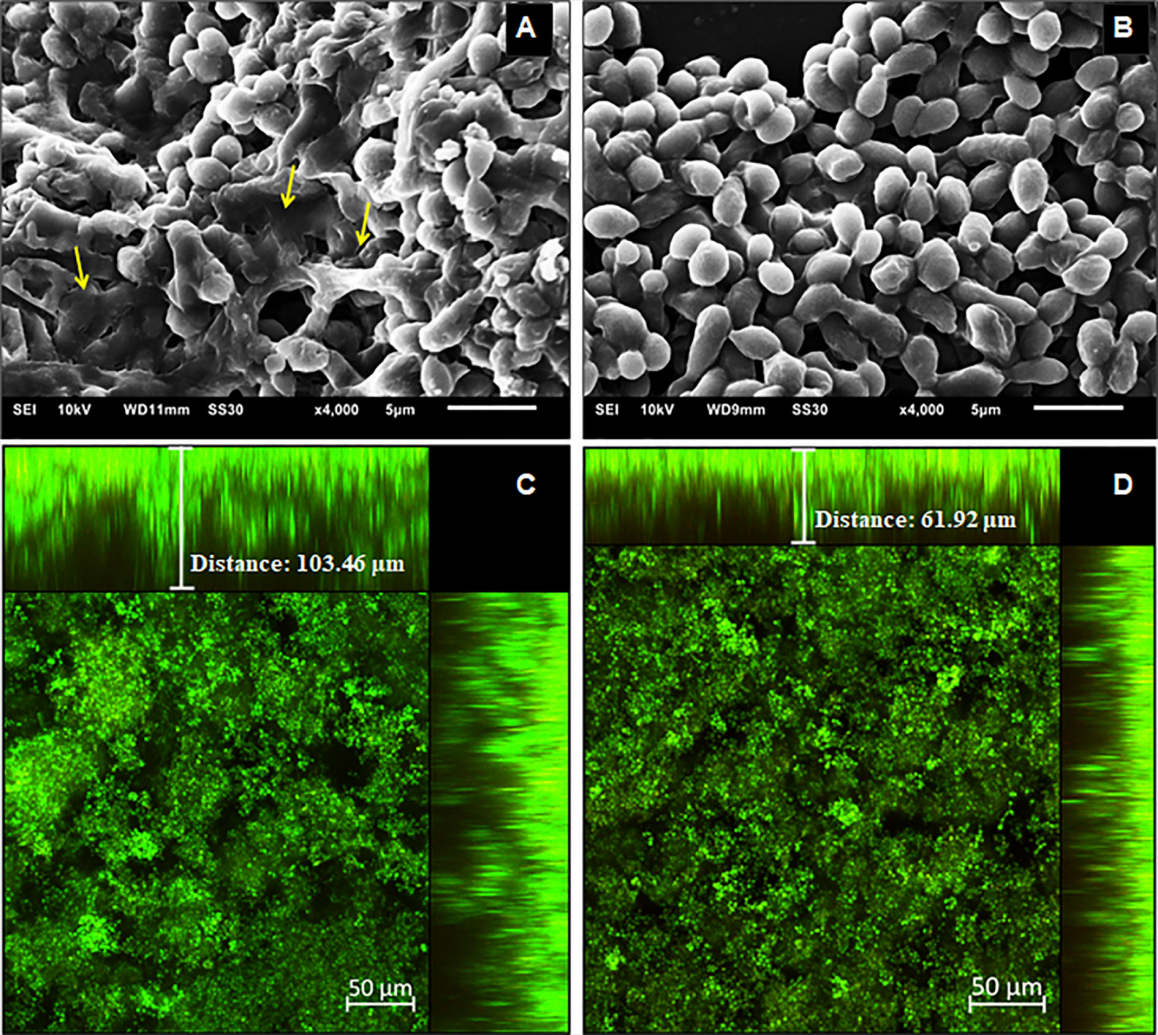
Structural analysis of the 144 h *H*. *capsulatum* EH-315 biofilms. Scanning electron microscopy (SEM) of *H. capsulatum* EH-315 non-treated **(A)** and treated **(B)** with Hsp60 mAb 7B6 at 4,000x. Confocal laser scanning microscopy (CLSM) biofilm images from *H. capsulatum* EH-315 were treated with Hsp60 mAb 7B6 **(D)** and control without treatment **(C)**. CLSM images comprising an orthogonal view of Z-stacks and 3D image of Z-stacks at 20x. The yellow arrows indicate the extracellular polymeric matrix (EPM).

**Figure 6:**
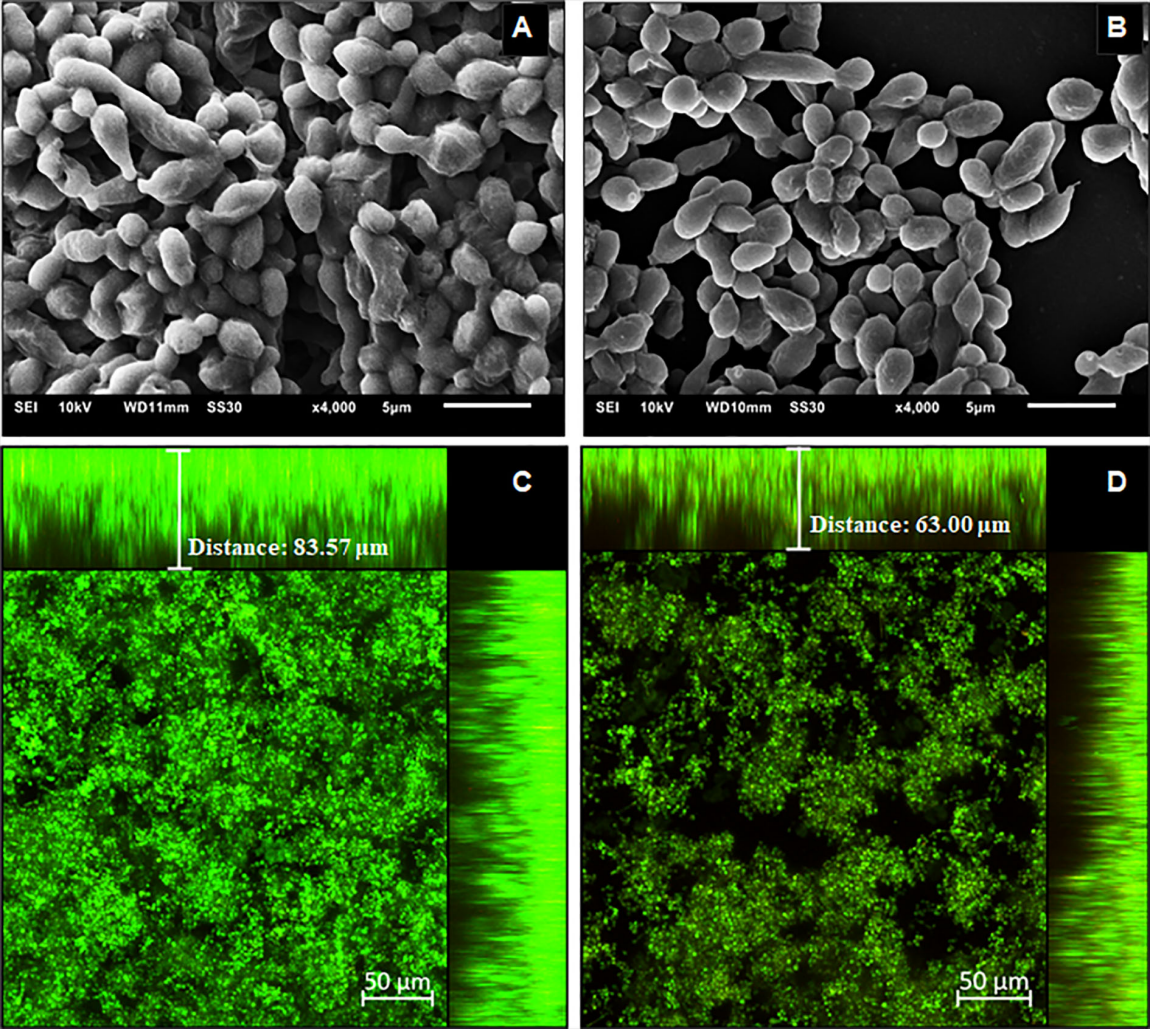
Structural analysis of the 144 h *H. capsulatum* G186A biofilms. Scanning electron microscopy (SEM) of *H. capsulatum* EH-315 non-treated **(A)** and treated **(B)** with Hsp60 mAb 7B6 at 4,000x. Confocal laser scanning microscopy (CLSM) biofilm images from *H. capsulatum* G186A treated with Hsp60 mAb 7B6 **(D)** and control without treatment **(C)**. CLSM images comprising an orthogonal view of Z-stacks and 3D image of Z-stacks at 20x.

To characterize the structure, density, and cell distribution of the biofilms formed after yeasts pre-treatment with Hsp60 mAb 7B6 and the control biofilms, SEM, and CLSM images were examined (**Figures 5** and **6**). The reduction of biomass content observed by the crystal violet staining could also be visually observed by SEM and CLSM.

The SEM data provided useful information on the cell morphology presented in the biofilm structure of both control and pre-treated biofilms. The biofilms of EH-315 (**Figure 5A**) presented a high amount of yeasts embedded in an EPM. The biofilms formed after pre-treatment of EH-315 with Hsp60 mAb 7B6 (**Figure 5B**) resulted in a visual reduction of the number of yeasts and the presence of the EPM.

Compared to the EH-315 strain, G186A formed a biofilm with visually less EPM (**Figure 6A**). However, the biofilms formed after the pre-treatment of G186A with Hsp60 mAb 7B6 also presented reduce in the total yeast distribution (**Figure 6B**).

CLSM showed that the biofilms of both EH-315 (**Figure 5D**) and G186A (**Figure 6D**) strains formed with pre-treated yeasts are thinner them their respective control (**Figures 5C** and **6C**, respectively). To the EH-315 strain, the control biofilm presented a thickness of 103.5 μm (**Figure 5C**), while the pre-treated biofilm presented 61.9 μm (**Figure 5D**). To the G186A strain, the control biofilm presented 83.6 μm of thickness (**Figure 6C**), whereas the pre-treated biofilm presented 68.0 μm (**Figure 6D**).

### Blockage of *H. capsulatum* Hsp60 Impairs the Survival of Infected *G. mellonella*

**Figure 7:**
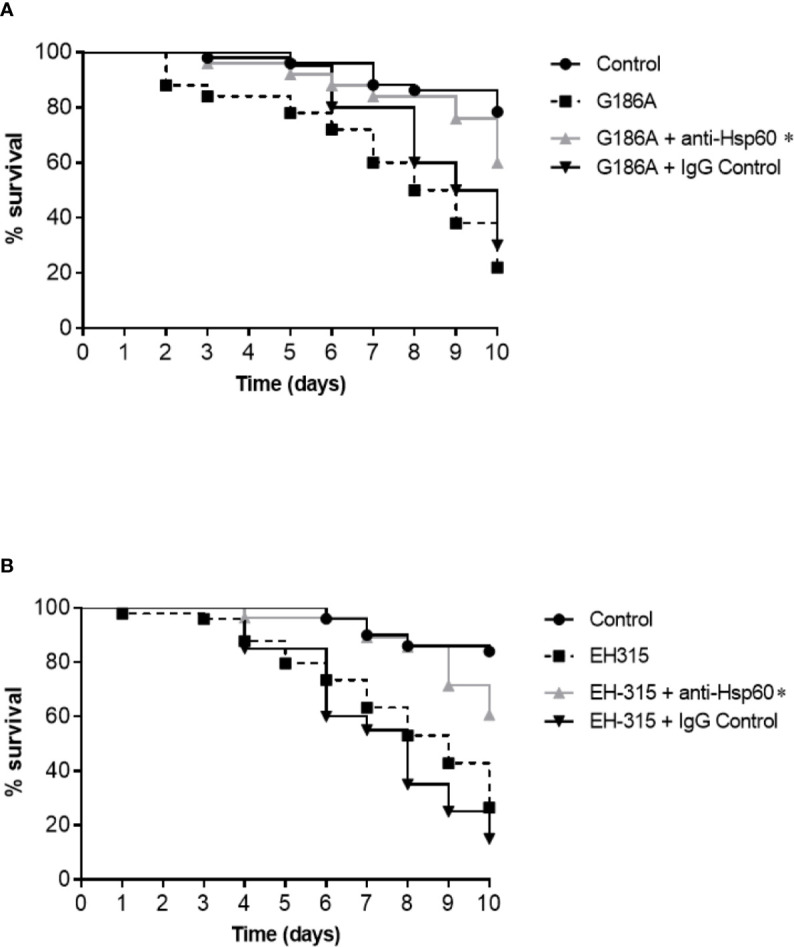
Influence of Hsp60 in the virulence of *H. capsulatum* G186A **(A)** and EH-315 **(B)** strains using *G mellonella* as a model. Data are representative of three independent experiments. The groups of larvae infected with untreated yeasts are represented by the black dashed lines, larvae infected with yeasts treated with control IgG are represented by the black lines and the groups of larvae infected with yeasts treated with Hsp60 mAb 7B6 are represented by the gray lines. The pre-treatment of both *H. capsultaum* strains with Hsp60 mAb 7B6 significantly increased the survival of *G. mellonella* compared with non-treated yeasts and treated with unspecific IgG. *p < 0.05.

An *in vivo* assay using the *G. mellonella* model was also performed to address whether Hsp60 exerted influence on larvae infected with *H. capsulatum*. First, the inoculation of both G186A and EH-315 strains led to a significant reduction in the larvae survival rate compared to the uninfected control (p < 0.05; **Figure 7**). We also observed that the G186A strain was slightly more virulent than the EH-315 strain since the larval survival rate on the tenth day was 22 and 26.5% after infection of each strain, respectively. Second, the blockage of Hsp60 with the Hsp60 mAb 7B6 before infection of *G. mellonella* larvae resulted in a significant increase (p < 0.05) of the larvae survival with rates on the tenth day of 60 and 60.7% for G186A (**Figure 7A**) and EH-315 (**Figure 7B**) strains, respectively, compared to larvae infected with untreated fungi. On the other hand, the pre-treatment of both strains with control IgG did not alter the survival curve of larvae compared to those of untreated fungi.

## Discussion

Heat shock proteins (Hsps), ubiquitously present in cells, are molecular chaperones conserved between microorganisms, being grouped according to their molecular mass and degree of amino acid homology. This nomenclature comes from the characteristic of being inducible through a rapid elevation in temperature. Currently, it is known that Hsps shown changes in expression profile in response to a range of stimuli, not always restricted to temperature, but also starvation, pH, pharmacological agents, and oxidative/osmotic stress (Burnie et al., 2006; Rappleye and Goldman, 2006).

A 60 kDa Hsp, known as Hsp60, is one of the most-characterized molecules on the surface of *H. capsulatum* G217B strain (Long et al., 2003; Guimaraes et al., 2009, Guimaraes et al., 2011a). Besides being described as a molecular chaperone and enhance cellular survival under physiological stress (Kubota et al., 1995, Guimaraes et al., 2011a), Hsp60 also interacts specifically with CD11b/CD18 (CR3) on macrophages surface, facilitating the uptake of yeast cells by these phagocytes, where the yeasts can survive and replicate (Long et al., 2003; Guimaraes et al., 2009), and also possess both antigenic (Gomez F. J. et al., 1991) and immunogenic activities (Gomez A. M. et al., 1991), highlighting it’s importance as a target for diagnostic and therapeutic approaches.

Moreover, the *H. capsulatum* cell surface presents several proteins that participate in host-pathogen interactions (Batanghari et al., 1998; Long et al., 2003; Bohse and Woods, 2007), sensing the environment (Isaac et al., 2013; DuBois et al., 2016) and defending the fungus against oxidative stress (Youseff et al., 2012; Holbrook et al., 2013). However, only a few have been tested for virulence roles in all strain backgrounds. Most adhesins used by *Histoplasma* to gain entry into host macrophages have only been determined for G217B strain, representative of chemotype I (Long et al., 2003; Gomez et al., 2008).

*Histoplasma capsulatum* strains can be divided into two chemotypes based on cell wall composition. Chemotype I lacks cell wall α-(1,3)-glucan and is represented by the G217B strain. Chemotype II, represented by G186A strain, contains a layer of α-(1,3)-glucan that masks immunostimulatory β-(1,3)-glucans from detection by the Dectin-1 receptor on host phagocytes (Rappleye et al., 2007). The α-(1,3)-glucan cell wall component is essential for chemotype II *H. capsulatum* virulence (Rappleye et al., 2004). In contrast, even without α-(1,3)-glucan, chemotype I remain fully virulent *in vivo* (Mayfield and Rine, 2007). Posteriorly Edwards et al. (2011) demonstrated that in the chemotype I the β-(1,3)-glucans are also not fully exposed and it is related to the growth phase, with more exposition during the exponential growth, and therefore allowing some interaction with Dectin-1. But in the stationary phase, the yeasts are practically undetectable, suggesting a particular mechanism to hide β-(1,3)-glucans in chemotype I.

Given the previously important roles described for Hsp60 in *H.* capsulatum, here we decided to advance and contribute to a better understanding of *H. capsulatum* Hsp60 regarding the biofilm scenario and host-pathogen interaction with the non-conventional model *G. mellonella.* For that purpose, we used a monoclonal antibody (7B6) to block the Hsp60. The biofilms of two *H. capsulatum* strains were characterized by metabolic activity, biomass content, and images from scanning electron microscopy (SEM) and confocal laser scanning microscopy (CLSM). The *G. mellonella* infection was assessed by the establishment of the survival curve.

According to the metabolic activity, the growth stage of G186A and EH-315 biofilms comprises the period of 72 to 144 h, with an increase in metabolic activity. After 144 h, both strains produced consistent and mature biofilms, reaching the stationary phase between 144 and 168 h. Compared to other fungal pathogens, as *Candida* spp. (Sánchez-Vargas et al., 2013; Chandra and Mukherjee, 2015) and *Cryptococcus neoformans* (Martinez and Casadevall, 2007), our results showed that *H. capsulatum* exhibits a slower growth as a biofilm structure, similar to those found on *Paracoccidioides brasiliensis* (Sardi et al., 2015) and *Sporothrix schenckii* complex (Brilhante et al., 2018) biofilms. Also, EH-315 formed a more robust biofilm compared to G186A, corroborating with the findings of Gonçalves et al. (2020).

The blockage of Hsp60 was effective to reduce the metabolic activity and biomass of the biofilms from both *H. capsulatum* strains. Furthermore, the biofilms of cells treated with the antibody were thinner as well as presented a lower amount of cells and extracellular matrix compared to its non-treated controls, revealing the potential role of the Hsp60 in cell-cell or cell-surface adhesion, increasing the importance of this protein as a virulence factor of *H. capsulatum.* Guimaraes et al. (2011b) showed that the Hsp60 mAb 7B6 reduces the formation of *H. capsulatum* aggregates. This antibody has an inconsistent impact on agglutinate charge resulting in reduced cell-to-cell interaction leading to a reduced *H. capsulatum* agglutination (Guimaraes et al., 2011b). In this way, we hypothesize that a reduced cell-to-cell interaction caused in *H. capsulatum* by the treatment with the Hsp60 mAb 7B6 can contribute to the reduction of the biofilm formation observed in our study.

*H. capsulatum* Hsp60 has never been related to the adherence of the fungus to abiotic surfaces nor implicated in the biofilm structure. However, antibodies specific to *Histophilus somni* Hsp60, an opportunistic pathogen that causes respiratory, genitourinary, and generalized infections in cattle, also prevented biofilm formation *in vitro* (Zarankiewicz et al., 2012).

Most *H. capsulatum* studies focus on phagocytosis or immune response. However, the demonstration that *H. capsulatum* yeasts can form biofilm *in vitro* and also can adhere to pneumocytes (Pitangui et al., 2012), cryopreserved bat organs (Suarez-Alvarez et al., 2010), human endothelium (Ledtke et al., 2012), and prosthetic valves (Alexander et al., 1979; Lorchirachonkul et al., 2013), draw attention to the importance of *H. capsulatum* adherence to the colonization and dissemination of the fungus. Furthermore, the susceptibility of *H. capsulatum* biofilms to amphotericin B and itraconazole was reduced comparing to the planktonic growth (Brilhante et al., 2015), highlighting the importance of studying the *H. capsulatum* biofilm structure.

Nonetheless, fungal biofilms are an important clinical problem associated with significant rates of antifungal resistance, disease persistence, and an increase of mortality index (Uppuluri et al., 2010; Kollef et al., 2012; Kowalski et al., 2019).

Despite the critical role that biofilms play in the course of the disease and colonization of host tissues, many basic aspects of development and organization, such as the initial steps of adhesion to the substrate, remain inconclusive. Some families of genes have already been described as important for this adhesion process, for example, the ALS, HWP, and IFF/HYR described to *Candida albicans*, that encodes several proteins responsible to facilitate cell-cell adhesion and adhesion of *C. albicans* on abiotic surfaces (Chandra et al., 2001; Hoyer et al., 2008; Ene and Bennett, 2009; Kempf et al., 2009). A marked reduction in total biofilm biomass has been shown in adhesin knockouts *C. albicans* strains, including *Δhwp2*, *Δhyr1*, and *Δals1/Δals3* double deletion mutants (McCall et al., 2019), revealing the importance of initial adhesion for the development of biofilm biomass.

Although remarkable in *C. albicans* biofilms, in which ALS genes exhibited increased expression (O’Connor et al., 2005), there is a lack of studies regarding the presence or impact of the adhesins on biofilms among the dimorphic fungi. Sardi et al. (2015) showed the up-regulation of GP43 and GAPDH in the biofilm of *P. brasiliensis*. GP43 is an important adhesin described in *Paracoccidioides* adhesion to matrix components (Vicentini et al., 1994), while GAPDH appears to impact the adhesion processes of both *Paracoccidioides* and *Candida* spp. (Gozalbo et al., 1998; Barbosa et al., 2006).

Notably, adhesins play an important role in biofilm formation and, possibly, the reduction of biomass, concomitantly with the reduction of metabolic activity, thickness, and presence of EPM in *H. capsulatum* biofilms formed after blocking the Hsp60 protein, suggest that this protein can also act on the adhesion to the substrate or in cell-cell adhesion, contributing to the establishment of *H. capsulatum* biofilms.

We also evaluated the role of Hsp60 of *H. capsulatum* in an invertebrate animal model, *G. mellonella*. The infection of the larvae with *H. capsulatum* was firstly tested by Thomaz et al. (2013), which used the G184ARAR (ATCC 26027) and ATCC G217B (ATCC 26032) strains to compare their virulence using different inoculum concentrations and temperatures (25 and 37 °C). Although we used other strains in this study (G186A and EH-315), the profile of the survival curve infected with 1x10^6^ yeast/larvae was similar to those, resulting in survival rates of about 20% at the end of 10 days at 37 °C in both studies. Interestingly, when the Hsp60 of G186A and EH-315 strains were blocked by 7B6 mAb before the larval infection, a significant increase in survival rate around 60% was observed.

Our findings reinforce that Hsp60, and its blocking by different antibodies, have different functions, which vary depending on the conditions and models tested. For example, Guimaraes et al. (2009) showed that the pre-treatment of mice with different antibodies anti-Hsp60 followed 2 h later by infection of *H. capsulatum* yeast cells resulted in a distinct response profile. IgG1 (11D1) and IgG2a (12D3) mAbs significantly prolonged survival and reduced the fungal load of animals, while IgG2b (7B6) mAb was not protective. Furthermore, the use of other antibodies, with different epitopes regions, promoted an increase in the phagocytosis by macrophages *in vitro*, but 7B6 and 6B7 (which both comprise the same structural cleft region of Hsp60) did not increase the phagocytosis. Specifically for 7B6, the epitope region is between 353 and 413 aa. This region represents the structural cleft on Hsp60, one of the regions responsible for CR3 interaction (Habich et al., 2006; Guimaraes et al., 2009). The presence of the Fc region itself can promote phagocytosis, however, considering that it does not occur to treatment with 7B6 and 6B7 antibodies, in addition to the presence of the Fc portion, maybe the structural cleft of the protein must be available for full interaction with CR3. Based on this, we hypothesized that this structural cleft region might not be important in the fungus-hemocyte interaction of the *G. mellonella* model. Also, although many similarities between insect and mammalian immune cells are observed, some differences such as the absence of Fc receptor in *G. mellonella* hemocyte compared to macrophage is described (Browne et al., 2013).

Another important aspect of the interaction of the Hsp60 mAb 7B6 with *H. capsulatum* was explored recently by Burnet et al. (2020). Significant changes in the plasma membrane induced when *H. capsulatum* yeast cells are treated with this and other Hsp60 mAb were shown. In all the tested mAb, but especially the 7B6, these membrane changes were characterized by an increased level of ergosterol, lead to higher sensitivity to the antifungal drug amphotericin B (Burnet et al., 2020). This higher sensitivity caused by alterations in the plasma membrane induced by the antibody can also lead to a higher sensitivity of the fungi to the immune system of the *G. mellonella*, increasing larval survival, as observed in our study.

Thus, the sum of these features can promote a different *Histoplasma*-hemocyte interaction and phagocytosis can occur normally, and consequently, lead to the death of the fungus and increasing larval survival. However further studies are needed to support this hypothesis.

Because of the importance of the different yeast ligands and host receptors on the intracellular fate of *H. capsulatum* and also the importance of the biofilms as mentioned above, the knowledge of the surface molecules repertoire that engage host infections and fungal adhesion might contribute to a better understanding of *Histoplasma* cell biology and virulence, as well as providing news targets to a more broadly applicable alternative to conventional antifungals.

## Data Availability Statement

The original contributions presented in the study are included in the article/supplementary materials. Further inquiries can be directed to the corresponding authors.

## Author Contributions

NF, HD, and AF-A conceived the presented idea. NF, LO, and CD designed and performed the experiments. NF, CM, HD, and JS processed the experimental data and verified the analytical methods. NF, HD, JS, CM, LO, and CD wrote the manuscript and designed the figures. MT, MM-G, HD, and AF-A supervised the findings and the writing of this manuscript. All authors discussed the results and contributed to the final manuscript.

## Funding

This work was supported by the Fundação de Amparo à Pesquisa do Estado de São Paulo-FAPESP [16/11836-0 (AF-A), 2019/04882-4 (NF), 2015/14023-8 (HD), 2016/17048-4 (CM), 2017/06658-9 (JS), 2018/15877-9 (LO)], Programa de Apoio ao Desenvolvimento Científico da Faculdade de Ciências Farmacêuticas da UNESP-PADC, Coordenação de Aperfeiçoamento de Pessoal de Nível Superior—Brasil (CAPES), and Conselho Nacional de Desenvolvimento Científico e Tecnológico (CNPq).

## Conflict of Interest

The authors declare that the research was conducted in the absence of any commercial or financial relationships that could be construed as a potential conflict of interest.
